# Inside help from the microbiome

**DOI:** 10.7554/eLife.88873

**Published:** 2023-06-05

**Authors:** Sneha Agrawal, Nichole A Broderick

**Affiliations:** 1 Department of Biology, Johns Hopkins University Baltimore United States

**Keywords:** Lactiplantibacillus plantarum, symbiosis, cell wall, growth, microbiome, *D. melanogaster*

## Abstract

Elucidating the role of one of the proteins produced by *Lactiplantibacillus plantarum* reveals a new molecule that allows this gut bacterium to support the development of fruit fly larvae.

**Related research article** Nikolopoulos N, Matos R, Ravaud S, Courtin P, Akherraz H, Palussiere S, Gueguen-Chaignon V, Salomon-Mallet M, Guillot A, Guerardel Y, Chapot-Chartier MP, Grangeasse C, Leulier F. 2023. Structure-function analysis of *Lactiplantibacillus plantarum* DltE reveals D-alanylated lipoteichoic acids as direct cues supporting *Drosophila* juvenile growth. *eLife* **12**:e84669. doi: 10.7554/eLife.84669.

Whether an animal can grow and put on weight as a juvenile not only depends on the nutrients it receives, but also on the microorganisms that live inside its gut (Schwarzer et al., 2016). In fruit flies, for example, a broad range of microbes can support the growth of malnourished larvae (Keebaugh et al., 2018). A closer look at the mechanisms involved in these interactions has shown that certain gut bacteria increase the protein and moisture content of the food the insect consumes; however, it has also revealed that cell wall components such as peptidoglycan can trigger intestinal cells to produce proteases that help flies make the most of their nutrients (Erkosar et al., 2015; Lesperance and Broderick, 2020). It therefore remains unclear whether the microbiome supports growth by altering the nutritional quality of food or by directly influencing certain biological processes in the flies.

Previous studies have shown that certain strains of *Lactiplantibacillus plantarum*, a species of Gram-positive bacteria which colonize in the gut of fruit flies, can boost the growth of larvae raised with limited access to proteins (Shin et al., 2011; Storelli et al., 2011). This ability relies on a cluster of six co-regulated genes known as the *pbpX2-dltXABCD* operon; five of these genes (*dltX*, *dltA*, *dltB*, *dltC* and *dltD*) code for proteins that help to add the molecule D-alanine onto teichoic acids, the most abundant component in the cell wall of Gram-positive bacteria (Matos et al., 2017; Nikolopoulos et al., 2022).

Two types of teichoic acids exist: lipoteichoic acids, which are anchored in the cell membrane, and wall teichoic acids, which are attached to peptidoglycan. Together these complex polymers allow the bacteria to interact with their host in both beneficial and pathogenic ways (Atilano et al., 2010; Brown et al., 2013). In *L. plantarum*, the D-alanine esterification of the cell wall which is supported by the *pbpX2-dltXABCD* operon leads to intestinal cells producing peptidases that are important for growth (Matos et al., 2017). However, the role of the *pbpX2* gene in the operon has remained unclear. Now, in eLife, Marie-Pierre Chapot-Chartier, Christophe Grangeasse, François Leulier and colleagues — including Nikos Nikolopoulos, Renata Matos, Stéphanie Ravaud and Pascal Courtin as joint first authors — report additional insights into the protein coded by *pbpX2*, and the role of teichoic acids in the growth of fruit fly larvae (Nikolopoulos et al., 2023).

The team (who are based in various institutes in France and Japan) found that the enzyme *pbpX2* encodes does not participate in the maturation of peptidoglycan, as was previously expected. Instead, further analyses showed that it works alongside the Dlt proteins from the operon to add D-alanine onto the glycerol residues of lipoteichoic acids. These results led Nikolopoulos et al. to rename the protein ‘DltE’, and to consider D-alanylated lipoteichoic acids as a signal required for *L. plantarum* to support intestinal function and juvenile growth.

Experiments conducted on LpNC8, a strain of *L. plantarum* known to help larvae develop when nutrients are scarce, allowed Nikolopoulos et al. to better understand which cell wall components the bacteria need for their growth-boosting role. This showed that LpNC8 primarily produces glycerol residues for its lipoteichoic acids, but ribitol residues for its wall teichoic acids. In fact, unlike other *L. plantarum* strains, only lipoteichoic acids are D- alanylated in LpNC8 bacteria. This suggests a need to systematically analyze the composition of teichoic acids and their modifications across multiple *L. plantarum* strains to understand how these patterns correlate with the bacteria’s ability to support fly growth.

Finally, the team investigated the roles of different cell wall components in larval development by purifying them individually from LpNC8 bacteria or from a LpNC8 mutant strain lacking the operon. This demonstrated that, unlike wall teichoic acids or unaltered lipoteichoic acids, D-alanylated lipoteichoic acids are a necessary and sufficient cue for promoting larval growth and for inducing the production of proteases in the gut. However, the presence of both peptidoglycan fragments and D-alanylated lipoteichoic acids is needed for optimal growth, suggesting that the flies use independent, additive signals to boost the production of peptidases and overall juvenile development (Figure 1).

**Figure 1.**
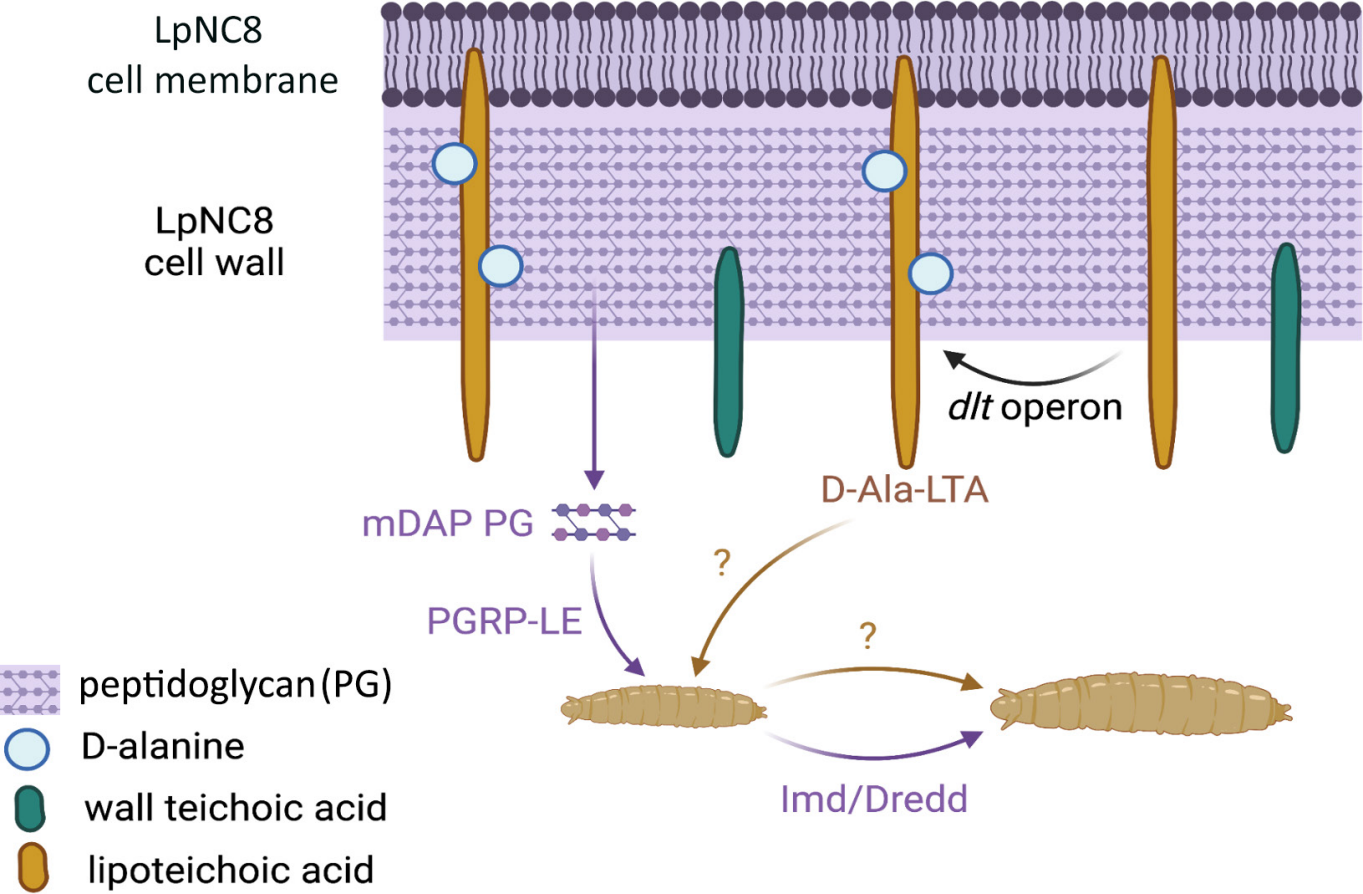
The recognition of certain bacterial cell wall components supports the growth of fruit fly larvae. Certain strains of the bacteria *Lactiplantibacillus plantarum*, such as LpNC8, can help the fruit fly larvae (bottom) in which they live to grow when nutrients are scarce. Various components of the *L. plantarum* cell wall (top) help with this process. For instance, fragments of peptidoglycan (mDAP PG) can be recognized by peptidoglycan recognition receptors (PGRP-LE) present at the surface of the intestinal cells of the fly; this, in turns, triggers a molecular pathway (Imd/Dredd) which leads to the production of proteases that help the larva grow. Nikolopoulos et al. show that other cell wall components known as D-alanylated lipoteichoic acids (D-Ala-LTA; yellow) also support protease production and larval growth through an independent, additional mechanism that remains unknown. They demonstrate that the addition of D-alanine (pale blue circles) onto lipoteichoic acids is supported by the proteins generated by the *pbpX2-dltXABCD* (or *dlt*) operon; this includes the protein encoded by the *pbpX2* gene, which they rename DltE. LpNC8 is unique in that D-alanine can only be added to its lipoteichoic acids, but not other similar polymers known as wall teichoic acids (green).

It remains unclear how D-alanylated lipoteichoic acids in *L. plantarum* manage to reach cells lining the midgut of *Drosophila*. Nikolopoulos et al. propose that the molecules may be released inside micro-vesicles that the intestinal cells then capture through endocytosis. The signaling pathways by which D-alanylated lipoteichoic acids induce intestinal proteases also remains to be identified, but they are probably separate from the molecular cascade elicited by peptidoglycans and other cell wall components. Overall, these results add to increasing evidence demonstrating the breadth and diversity of the bacterial components that underlie fly-microbiome interactions. As *L. plantarum* also supports growth in mice, this study opens new, exciting avenues of research into the role of host bacteria in the development of mammals and other animals.
